# Exercise suppresses tumor growth independent of high fat food intake and associated immune dysfunction

**DOI:** 10.1038/s41598-022-08850-5

**Published:** 2022-03-31

**Authors:** Pernille Hojman, Rikke Stagaard, Emi Adachi-Fernandez, Atul S. Deshmukh, Andreas Mund, Caroline H. Olsen, Lena Keller, Bente K. Pedersen, Julie Gehl

**Affiliations:** 1 Centre for Physical Activity Research (CFAS) and Centre of Inflammation and Metabolism (CIM), Rigshospitalet, University of Copenhagen, Copenhagen, Denmark; 2 Novo Nordisk Foundation Center for Protein Research, Clinical Proteomics, Faculty of Health and Medical Sciences, University of Copenhagen, Copenhagen, Denmark; 3 Department of Pathology, Rigshospitalet, Copenhagen, Denmark; 4 Department of Biomedicine (DBM), University Hospital Basel, Basel, Switzerland; 5 Center for Experimental Drug and Gene Electrotransfer (C*EDGE), Department of Oncology and Palliative Care, Zealand University Hospital, Roskilde, Denmark; 6 Department of Clinical Medicine, Faculty of Health and Medical Sciences, University of Copenhagen, Copenhagen, Denmark

**Keywords:** Oncology, Cancer metabolism

## Abstract

Epidemiological data suggest that exercise training protects from cancer independent of BMI. Here, we aimed to elucidate mechanisms involved in voluntary wheel running-dependent control of tumor growth across chow and high-fat diets. Access to running wheels decreased tumor growth in B16F10 tumor-bearing on chow (− 50%) or high-fat diets (− 75%, *p* < 0.001), however, tumor growth was augmented in high-fat fed mice (+ 53%, *p* < 0.001). Tumor growth correlated with serum glucose (*p* < 0.01), leptin (*p* < 0.01), and ghrelin levels (*p* < 0.01), but not with serum insulin levels. Voluntary wheel running increased immune recognition of tumors as determined by microarray analysis and gene expression analysis of markers of macrophages, NK and T cells, but the induction of markers of macrophages and NK cells was attenuated with high-fat feeding. Moreover, we found that the regulator of innate immunity, ZBP1, was induced by wheel running, attenuated by high-fat feeding and associated with innate immune recognition in the B16F10 tumors. We observed no effects of ZBP1 on cell cycle arrest, or exercise-regulated necrosis in the tumors of running mice. Taken together, our data support epidemiological findings showing that exercise suppresses tumor growth independent of BMI, however, our data suggest that high-fat feeding attenuates exercise-mediated immune recognition of tumors.

## Introduction

Exercise training has consistently been shown to decrease the risk of cancer development and progression. Solid epidemiological data demonstrate that physical activity significantly reduces the risk of 13 out of 26 cancers both for individuals who are overweight/obese and normal-weight^1^. Moreover, physical activity after a cancer diagnosis is associated with a lower risk of disease recurrence and improved overall survival^2–4^. In parallel, preclinical studies have demonstrated a direct suppression of tumor growth and disease progression by exercise training across numerous different rodent tumor models^5,6^. These mechanistic studies have pointed to a pivotal role of exercise and epinephrine-dependent immune cell mobilization and regulation of intratumoral immune cell infiltration in the exercise-mediated suppression of murine tumors^7,8^.

In contrast, obesity is associated with an increased risk of cancer and poor survival across a number of cancer diagnoses^9^. While physical activity and exercise training can reduce body weight and adiposity, the protective effect of exercise can not solely be ascribed to improved weight control. In fact, the protection offered by exercise training extends across all categories of BMI^1^. A prevalent hypothesis has proposed that exercise may reduce the risk of cancer through an exercise-dependent regulation of common cancer risk factors, including sex hormones, insulin, insulin-like growth factor-1, and pro-inflammatory cytokines, which are all associated with obesity and adiposity^10,11^. The proposal of this causality has led to numerous large exercise intervention trials, aiming to reduce these risk factors through various structured training programs in women at high risk of developing breast cancer^12–14^. In general, modest reductions in systemic levels of sex hormones, insulin and pro-inflammatory cytokines were observed in these studies, and the reductions were largely dependent on loss of body weight during the exercise interventions^15^. In continuation, when the effect of exercise-mediated reductions in circulating risk factors are tested in vitro, the reductions in systemic risk factors did not translate into suppression of breast cancer cell proliferation^8,16^, questioning this causality. This implies that other factors may play an important role in the interplay between exercise and diet-dependent regulation of tumor growth. The ability of exercise to increase immune function could be such a factor, especially when considering that recent preclinical studies^17^ indicate that obesity can suppress antitumor immunity.

**Figure 1:**
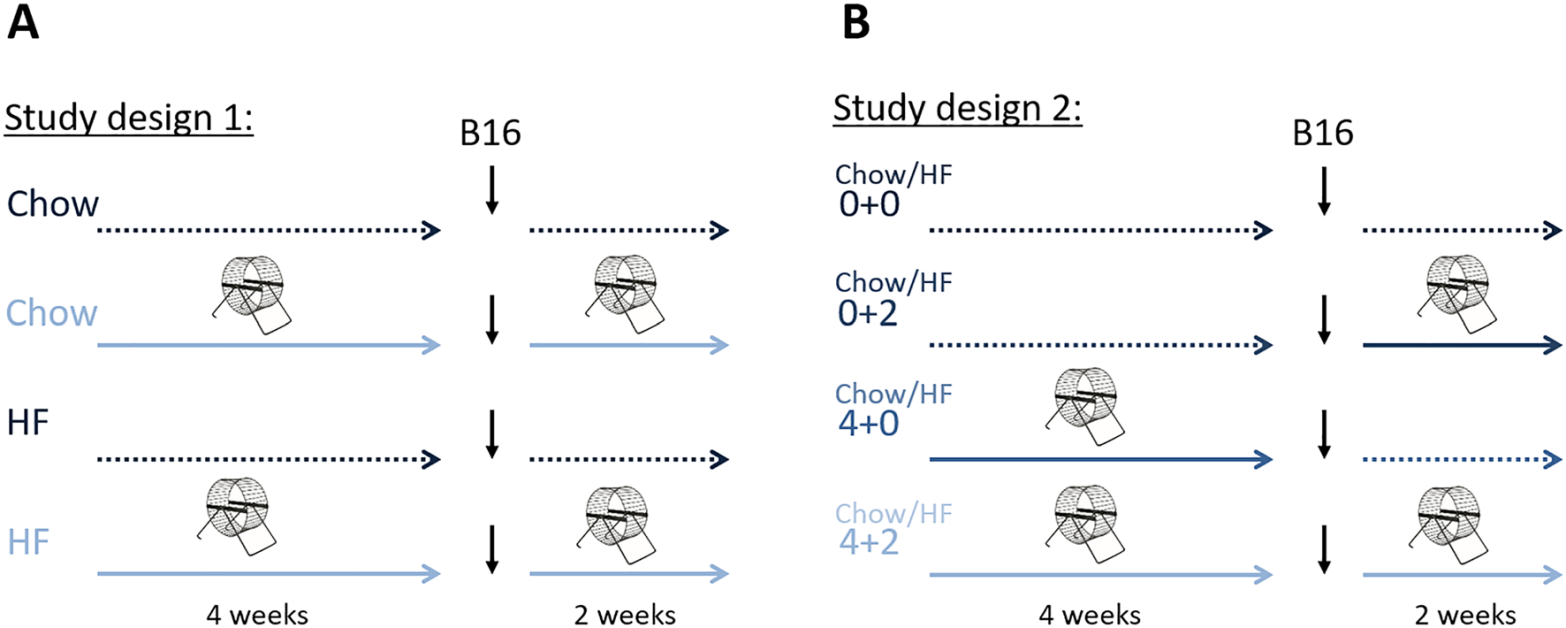
Illustrations of the two used study designs. (**A**) In study design 1, C57bl/6 mice were randomized to cages containing running wheels for 4 weeks prior to tumor inoculation and during tumor challenge (EX) or no running wheel (CON), and further randomized to chow or high-fat feeding (HF) (n = 12). (**B**) In a parallel study (study design 2), mice were randomized to cages containing running wheels for 4 weeks prior to tumor inoculation and during tumor challenge (4 + 2), 4 weeks just prior to tumor inoculation (4 + 0), 2 weeks during tumor challenge (0 + 2), or no running wheel (0 + 0) (n = 12), while either receiving a HF or standard chow diet.

**Figure 2:**
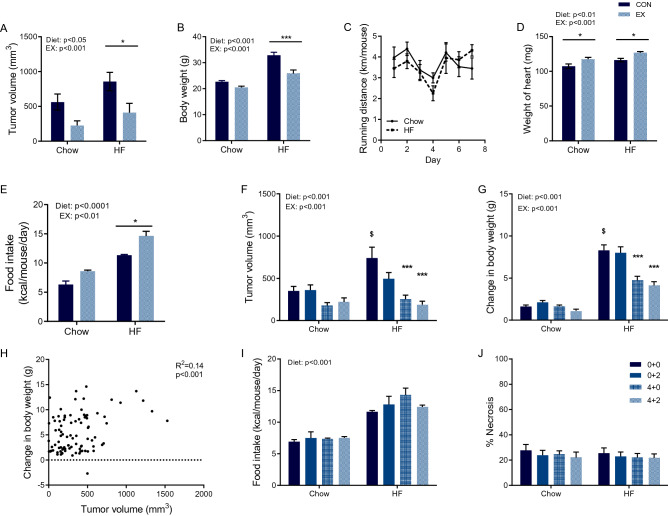
Effect of wheel running on B16 melanoma growth in mice fed chow or high-fat diet. C57bl/6 mice were randomized to cages containing running wheels for 4 weeks prior to tumor inoculation and during tumor challenge (EX) or no running wheel (CON), and further randomized to chow or high-fat feeding (HF) (n = 12, study design 1). In these mice, (**A**) tumor volume, (**B**) body weight, (**C**) running distance, and (**D**) weight of the heart were determined at termination, while (**E**) the average daily food intake was monitored both prior and after tumor induction. In a parallel study (study design 2), mice were randomized to cages containing running wheels for 4 weeks prior to tumor inoculation and during tumor challenge (4 + 2), 4 weeks just prior to tumor inoculation (4 + 0), 2 weeks during tumor challenge (0 + 2), or no running wheel (0 + 0) (n = 12). In these mice, (**F**) tumor volume, (**G**) change in body weight, (**H**) association between tumor volume and change in body weight, (**I**) the average daily food intake (including the period both prior and after tumor induction) and (**J**) percentage of necrotic area in B16 tumors from (2**F**) were determined. Statistical significance was determined by 2-way ANOVA with Tukey’s post hoc test (**A, B, D, E, F, G, I, J**) or linear regression analysis (**H**). *P* values for diet and EX describe the results of the 2-way ANOVA, while $ indicates statistical significance difference between Chow (0 + 0) and HF (0 + 0) at *p* < 0.05 and individual stars indicate difference from control (CON/0 + 0) group within each feeding group in the post hoc tests. **p < 0.01, ***p < 0.001.

To investigate the effect of high-fat feeding and adiposity on exercise-dependent regulation of tumor growth, we first explored the effect of voluntary wheel running on the growth of murine B16F10 melanoma tumors in C57Bl/6 mice (Fig. 1A). Wheel running significantly reduced tumor growth in both chow and high-fat fed mice (*p* < 0.001, Fig. 2A), but at the same time, the high-fat fed mice, in general, had larger tumors than the chow fed mice (*p* < 0.05, Fig. 2A). As expected, high-fat fed mice gained significantly more body weight than chow-fed mice (*p* < 0.001, Fig. 2B), while wheel running attenuated some of the diet-induced weight gain (*p* < 0.001). Of note, there was no difference in running distance between chow and high-fat fed mice (Fig. 2C), and running mice on either diet showed exercise adaptations, for instance in the form of cardiac hypertrophy (*p* < 0.001, Fig. 2D).

To decipher the importance of exercise dose, we provided running wheels for 0, 2, 4, and 6 weeks (Fig. 1B). The exercise-dependent suppression of tumor growth was dose-dependent with greater suppression of tumor growth after 4 and 6 weeks of wheel running than with 2 weeks (Fig. 2F). The timing of the exercise intervention could, however, also be of importance, as training prior to the tumor challenge had a larger effect. High-fat fed mice had an increased food intake (*p* < 0.001, Fig. 2E, 2I) and also gained significantly more body weight than chow-fed mice in this set-up (*p* < 0.001, Fig. 2G), and 4 and 6 weeks of wheel running attenuated this diet-induced weight gain by, respectively, 44% and 51% (*p* < 0.001, Fig. 2G). Hence, wheel running did not completely prevent high-fat feeding induced weight gain, but the suppression of tumor growth was to the same relative degree as observed in chow-fed mice. We explored the association between tumor volume and change in body weight and found a weak but significant correlation (R^2^ = 0.14, *p* < 0.001, Fig. 2H), which was primarily driven by the high-fat fed mice. In parallel, histological examinations of paraffin embedded tumors demonstrated about 20–30% necrosis within the tumors, but the extent of necrosis was not affected by neither wheel running nor high-fat feeding (Fig. 2J).

These experimental data are in line with current epidemiological evidence, demonstrating that exercise training and physical activity can prevent cancer and control tumor progression across all ranges of BMI^1^. Unfortunately, no blood lipids were measured during our studies, but it would have been very interesting to correlate a circulating lipidomic profile with the anti-tumor activity. Our data are also in accordance with another study in mice demonstrating that treadmill running could reduce intestinal polyp formation in Apc^Min/+^ mice fed a western-style diet^18^. In addition, we showed that higher volume of wheel running provides greater suppression of tumor growth in this voluntary wheel running model, suggesting a dose-dependent and timing effect of exercise on tumor growth. These results are in line with previous published data^7^, and could suggest that exercise not only have an effect on tumor growth, but also on tumor engraftment^7,19^. Furthermore, similar associations between larger volume of physical activity and greater protection against disease progression have been established by epidemiological studies^2,3^.

**Figure 3:**
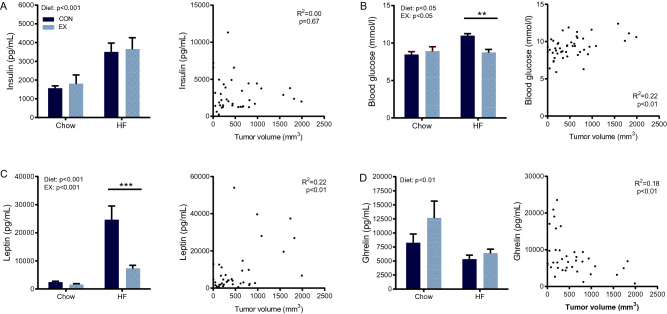
Tumor growth is correlated with systemic regulation of glucose, leptin and ghrelin. From mice in study design 1, serum concentrations and association to tumor volume of (**A**) insulin, (**B**) glucose, (**C**) leptin and (**D**) ghrelin were determined. Statistical significance was determined by 2-way ANOVA with Tukey’s post hoc test and linear regression analyses. P values for diet and EX describe the results of the 2-way ANOVA, while individual stars indicate difference from control (CON) group within each diet group. **p < 0.01, ***p < 0.001.

The obesity-driven tumor progression has been linked to regulation of systemic risk factors like insulin and low-grade inflammation^11^, thus we evaluated the levels of these systemic factors in mice randomized to no wheel running or 6 weeks of wheel running. High-fat feeding increased serum levels of insulin (*p* < 0.001, Fig. 3A), glucose (*p* < 0.05, Fig. 3B) and leptin (*p* < 0.001, Fig. 3C). Of these, wheel running significantly attenuated the serum levels of glucose (*p* < 0.05) and leptin (*p* < 0.001) (Fig. 3B, C), but had no effect on serum insulin levels. Moreover, serum glucose (R^2^ = 0.22, *p* < 0.01) and leptin (R^2^ = 0.22, *p* < 0.01) levels was associated with tumor volume, while no such association was observed for insulin (Fig. 3A–C). Contrarily, the appetite hormone ghrelin tended to increase with wheel running, yet this induction was mitigated by high-fat feeding (Fig. 3D). Serum ghrelin levels were negatively associated with tumor volume (R^2^ = 0.18, *p* < 0.01, Fig. 3D).

In this study, high-fat feeding induced serum insulin levels, but we did not observe any association between circulating insulin levels and regulation of tumor growth, which contrasts with previously published statements that insulin levels would promote tumor growth^11^. In contrast, tumor growth was positively associated with serum leptin levels, and negatively with serum ghrelin levels. Leptin has previously been shown to induce tumor growth and may be a mediating link between adiposity and enhanced tumor growth^20^. In contrast, little is known on the role of ghrelin in cancer control, even though we found an association, no causal relation can be concluded from this study. We also measured markers of systemic inflammation, but none of the pro-inflammatory cytokines TNF-α, IL-1β, or IL-6 were detectable in the serum (data not shown), possibly suggesting that low-grade inflammation played a minor role in this model.

**Figure 4:**
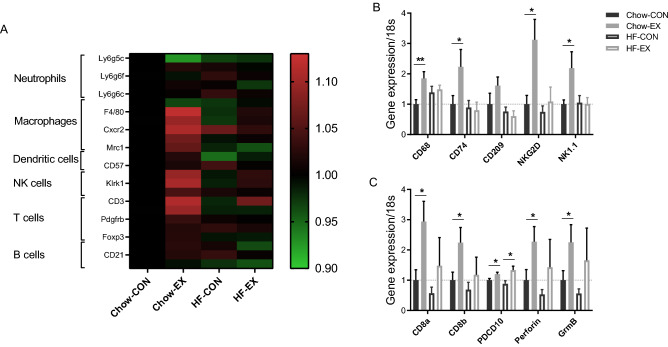
High-fat feeding suppresses exercise-mediated innate immune recognition. Expression of selected markers for different immune subsets by microarray analysis in C57bl/6 mice that were randomized to cages containing running wheels for 4 weeks prior to tumor inoculation and during tumor challenge (EX) or no running wheel (CON), and further randomized to chow or high-fat feeding (HF) (n = 5, study design 1) (**A**). Gene expression by PCR analysis of (**B**) markers of myeloid cells/macrophages and NK cells, and (**C**) markers and cytolytic enzymes of T cells (n = 9–12). Statistical significance was determined by 2-way ANOVA with Tukey’s post hoc test for each gene. Individual stars indicate difference from the control group. *p < 0.05, **p < 0.01.

Comprehensive preclinical studies have pointed to an exercise-dependent mobilization and activation of cytotoxic immune cells of both the innate and adaptive immune system as a key component in the exercise-dependent control of tumor growth, in particular in the B16F10 melanoma tumor model^5,7,21,22^. The present study extends these findings by addressing transcriptomic profiling of B16 tumors from chow and high-fat fed mice. In this study, we expanded upon our previously reported microarray analyses^7^ by also including the analyses of tumors from high-fat fed mice and comparing the results obtained with the chow fed and high-fat fed mice. We observed that markers of macrophages, NK cells, and T cells were upregulated by wheel running in chow-fed mice (Fig. 4A). The microarray analyses did not indicate that neutrophils, dendritic cells or B cells were regulated in the tumors by wheel running (Fig. 4A). Next, we validated these findings by PCR analysis and observed that the exercise-dependent enhanced expression of markers of myeloid cells/macrophages (CD68, CD74 and CD209) and NK cells (NKG2D and NK1.1) were indeed attenuated, when the mice were fed a high-fat diet (Fig. 4B). In contrast, the exercise-mediated induction of T cell markers was less affected by high-fat feeding (Fig. 4C).

In general, obesity is reckoned to impair the immune system^23^ and is associated with many immunological abnormalities, which may include a smaller pool of NK cells and CD8 + T cells and decreased dendritic cells function^24^. Moreover, obesity is associated with subclinical inflammation^25^, and this low-grade chronic inflammation is thought to derive from macrophages infiltrating the fat depots ^26^. In addition to the traditional pro-inflammatory cytokines, leptin is generally also considered a pro-inflammatory cytokine with stimulatory effects on macrophages, granulocyte chemotaxis, and Th17 proliferation^27^. Furthermore, chronic leptin stimulation can reduce NK cell cytotoxicity through impaired IFN-γ and perforin production^28,29^. Recently, obesity in humans and high-fat feeding in mice were demonstrated to paralyze NK cell metabolism and trafficking^30^. In particular, obesity and PPARα/δ signaling were shown to block trafficking of granules containing the cytotoxic enzymes Perforin and Granzyme B^30^. Besides the effect on NK cells, high-fat diet-induced obesity has also been shown to inhibit antitumor immune responses in several diet-sensitive cancer models. Especially, high-fat diet induced metabolic adaptions in the tumor microenvironment and reduced the infiltration and functionality of CD8 + T cells, potentially via the leptin-STAT3 axis and an altered utilization and oxidation of fatty acids^17,31^. These results are in line with our findings, as the exercise-mediated increase in expression of T cell markers was mitigated by high-fat feeding in both microarray and PCR analyses. The fact that we still see an effect of exercise on tumor growth in HF-fed mice, indicates that the tumor-reducing effect of exercise also involves other mechanisms than the noted modulation of an intra-tumor immune response. Potential mechanisms could be an exercise-mediated; (1) increase in peripheral lymphocyte proliferation as suggested by Dos Santos et al.^32^, (2) reduction in serum leptin levels, which may be mediating a link between obesity, T cell dysfunction, and enhanced tumor growth^31^, (3) reduction in the chronic low-grade inflammatory state that is often associated with obesity^32^, and lastly (4) reduction in the availability of the nutrients needed for tumor growth^33,34^.

**Figure 5:**
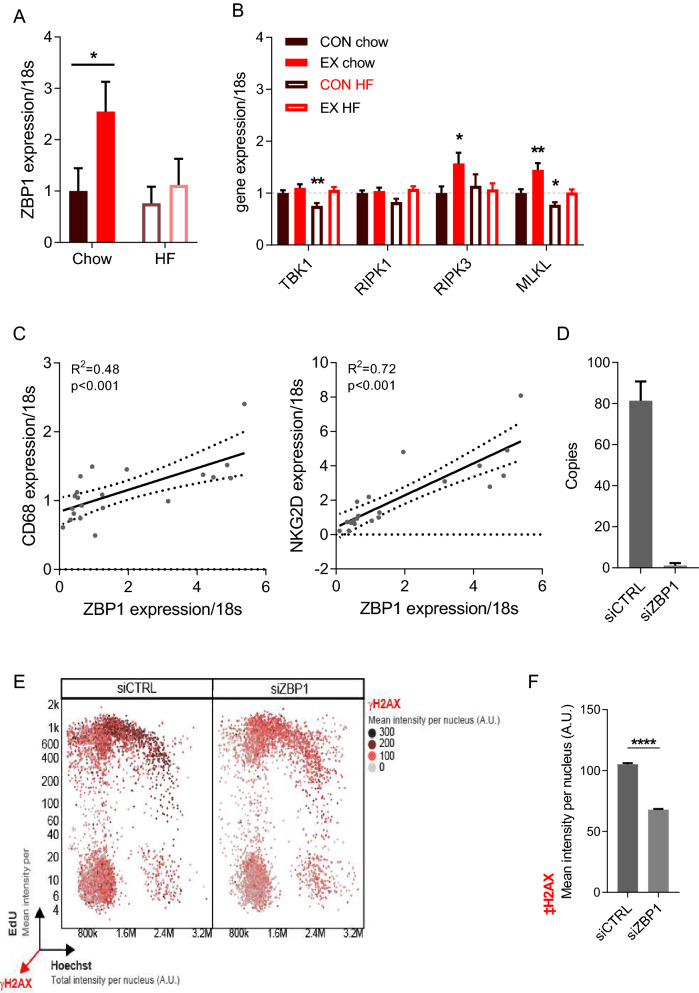
ZBP1 is induced by wheel running and associated with innate immunity. Expression of (**A**) ZBP1 and (**B**) ZBP1-related genes in B16 tumors from mice randomized to control (CON) or 6 weeks of wheel running (EX), and further randomized to chow or high-fat feeding (0) (n = 9–12, study design 1). (**C**) Association between intratumoral ZBP1 expression and markers of innate immune cells, i.e. CD68 and NKG2D. (**D**) Verification of ZBP1 knock-down (siZBP1) after siRNA transfection in B16 cells. (**E**) High-content imaging of cell cycle distribution in ZBP1 KO B16 cells obtained with quantitative image-based cytometry (QIBC). The intensity of the red color depicts the levels of γH2AX expression. (**F**) Quantification of the γH2AX impaired expression upon ZBP1 silencing. Statistical significance was determined by 2-way ANOVA with Tukey’s post hoc test, multiple *t*-testing, linear regression analyses, and Mann–Whitney tests. Individual stars indicate difference from the CON chow group. *p < 0.05, **p < 0.01, ****p < 0.0001.

Our microarray screening revealed an interesting target, namely ZBP1, which is a cytosolic DNA-sensing molecule that upon stimulation can initiate either cellular necroptosis or innate immunity^35^. PCR validation showed that intratumoral ZBP1 increased 2.5-fold (*p* < 0.05) by wheel running in the chow-fed group, while this induction was blunted in high-fat fed mice (Fig. 5A). Recent studies have highlighted a role of ZBP1 in inducing necroptosis through the RIPK pathway. Thus, we investigated if other factors of this pathway were regulated by 6 weeks of wheel running (Fig. 5B). Wheel running increased intratumoral Ripk3 expression by 53% (*p* < 0.05) and Mlkl by 45% (*p* < 0.01), and as for ZBP1 this exercise-mediated induction was blunted when the mice were placed on a high-fat diet (Fig. 5B). Of note, we observed no regulation of Tbk1 and Ripk1 in the B16F10 tumors by wheel running (Fig. 5B). Instead, the expression pattern of ZBP1 might be linked to the exercise-induced innate immune cell recognition, and in accordance we demonstrated significant association between ZBP1 expression with CD68 (R^2^ = 0.48, *p* < 0.001) and NKG2D (R^2^ = 0.72, *p* < 0.001) expression in the B16F10 tumors (Fig. 5C), suggesting that ZBP1 may be involved in the attraction of innate immune cells.

To investigate the second role of ZBP1, namely induction of necroptosis, we knocked down ZBP1 in B16F10 tumor cells (Fig. 5D) and evaluated their cell cycle distribution and cellular stress levels, as monitored by quantitative image-based cytometry (QIBC) and γH2AX induction (Fig. 5E). When comparing ZBP1-depleted cells with control cells, we did not observe any regulation of cell cycle arrest. Nonetheless, B16F10 cells treated with ZBP1 siRNA showed significantly reduced γH2AX levels (Fig. 5E, F), suggesting an altered DNA damage response and potential role of ZBP1 in reflecting cellular stress in these cells.

ZBP1 acts as an innate immune sensor and its functions include induction of Type I IFN immunity through proteins such as TBK1 and IRF3, as well as induction of necroptosis^35^. Together our results suggest that ZBP1-dependent necroptosis plays a minor role in the exercise-mediated control of tumor growth, in contrast ZBP1 could be linked to the regulation of the exercise-dependent induction of the innate immunity, as evidenced by a tight correlation to myeloid and NK cell markers in the B16F10 tumors, and the blunted response to wheel running during high-fat feeding. ZBP1 is unlikely to be the only molecule of interest in these changes, but we decided to investigate this particular molecule further based on the array analysis.

In conclusion, voluntary wheel running suppresses tumor growth across chow and high-fat diets, while high-fat diet independently enhances tumor growth. These data are in line with the epidemiological findings demonstrating that exercise can decrease the risk of cancer and cancer-specific mortality, independent of BMI, while obesity alone promotes cancer. Our data indicate that increased leptin levels but not insulin, is associated to increased tumor growth. Furthermore, our data show that high-fat feeding attenuates exercise-induced innate immune recognition of tumors, linking obesity and impaired immune function to enhanced tumor growth during high-fat feeding.

## Material and methods

### Animals, diets and exercise intervention

#### Mouse models and exercise interventions

All animal experiments were conducted in accordance with the recommendations of the European Convention for the Protection of Vertebrate Animals used for Experimentation and after permission from the Danish Animal Experiments Inspectorate. The studies were compliant with the ARRIVE guidelines. Mice were placed in standard housing cages and maintained in a thermo-stated environment under a 12-h light/dark cycle with free access to drinking water and food (respectively normal chow, 2844 kcal/kg, 4% crude fat, Altromin pellets, Spezialfutter-Werke, Germany or high-fat diet: 5240 kcal/kg, 34.9% crude fat; Research Diets, Bomholtgaard, Denmark).

Female C57BL/6 mice were bred in-house (breeding pairs were obtained from Harlan). For all studies, mice were randomized to chow or high-fat feeding (HF) at 8–12 weeks of age, and then further randomized to cages containing running wheels as a model of voluntary exercise or no running wheel (n = 12). For the exercise intervention, two different study designs were used. Either the exercising mice had access to running wheels throughout the study (study design 1, Fig. 1A) or before tumor cell inoculation and/or during the tumor challenge (study design 2, Fig. 1B)*.* As previously described^7^, to avoid isolation-induced stress, the mice were held with three mice in each cage and more than one mouse could run in the wheel at the same time. The total running distance was evaluated by the placement of bicycle computers on the running wheels. For all the above interventions, running distance and food intake were monitored. The mice were subcutaneously inoculated in the flank with 2 × 10^5^ B16F10 melanoma cells (cells obtained from ATCC), which had been tested by RapidMap27 (Taconic) to ensure the purity of the cells. Tumor establishment and growth was measured every second day by external caliper measurements. At termination, tumor and heart were excised, the heart weighed, and the tumor measured with a caliper. Tumor volume was determined by using the calculation V = d1 * d2 * d3 * π/6, where d is the diameter of the tumor^33^.

### Molecular analyses

#### Blood sampling and ELISA

Blood samples were collected at euthanasia by decapitation. Blood glucose levels were determined by PrecisionX glucometer (Abbott), while additional blood was collected, processed to serum and frozen at – 80°. Serum insulin, leptin, and ghrelin levels were determined by Luminex 9-plex assay according to the manufacturer’ directions.

#### Microarray data

We utilized our previously published microarray dataset^7^ on B16F10 tumors derived from mice randomized to wheel running deposited in NCBI’s Gene Expression Omnibus (GEO) with concurrent chow or high-fat feeding. The data from chow fed mice are accessible through GEO series accession number GSE62628.

#### RNA isolation and PCR

As described by Pedersen et al.^7^, total RNA was isolated from frozen tumor samples by tissue homogenization and RNA extraction using the TRIzol method (Invitrogen). Purity and quantity of the isolated RNA were determined by a Nanodrop spectrophotometer (Thermo Scientific). Total RNA (250 ng) was reversely transcribed into complementary DNA (cDNA) using the High Capacity cDNA Reverse Transcription kit with random hexamer primers (Applied Biosystems). All amplifications were carried out in a final volume of 10 µl with 3 µl cDNA and monitored in real time using the WiiA7 real-time PCR machine (Applied Biosystems) with SYBR green as a fluorescence marker (SYBR®Green PCR Master Mix, Applied Biosystem) using sequence specific primers. A heating dissociation step was added after each terminated PCR reaction to check for any potential unspecific PCR products. Quantification was performed by normalizing to standard curves for each gene. The target genes are presented as their ratio to ribosomal 18S RNA.

#### Immunofluorescence (IF)

As described by Coscia et al. and Somyajit et al.^36,37^, transfections of siRNA duplexes were achieved with Lipofectamine RNAiMAX (Thermo Fisher Scientific) at a final concentration of 5 nM for 72 h. For ZBP1 (58203) and respective control (001) siRNA pool were obtained from siTOOLS BIOTECH. After 72 h knockdown, cells grown on cover slips were first incubated with 5-ethynyl-2 deoxyuridine (EdU) for 20 min, then fixed for 10 min at room temperature with 4% paraformaldehyde and washed three times with PBS. Cells were then permeabilized with PBS/0.5% Triton-X for 30 min and blocked for 30 min in blocking buffer (3% BSA in PBS). Cells were then stained with an EdU labeling kit (Life Technologies) and incubated with primary antibody (γH2AX-Ser139, clone JBW301, Millipore 05-636-I) for 1 h in PBS/1% BSA in at RT. Following three washes, cells were incubated with secondary antibody, including Hoechst 33342 (diluted in PBS/1% BSA) for 1 h at room temperature. Slides were mounted with Mowiol 4-88.

#### Quantitative image-based cytometry (QIBC)

QIBC was performed as previously described^36–39^. In brief, images were acquired with a ScanR high-content microscope (Olympus) with wide-field optics, a 10×, 0.4-NA (UPLSAPO 10×) dry objective, a quadruple-band filter set for DAPI, FITC, Cy3, and Cy5 fluorescent dyes, a MT20 Illumination system and a digital monochrome Hamamatsu C9100 electron-multiplying CCD camera. Olympus ScanR software was used for automated images acquisition. Typically, 25 images were acquired containing at least 4000 cells per condition. Identical settings were applied to all samples using non-saturated conditions for the different channels. Images were processed and analyzed with ScanR analysis software. Data were then exported and analyzed with TIBCO Software. This software was used to quantify absolute, and median values in cell populations and to generate all color-coded plots. Within one experiment, same cell numbers were compared for the different conditions.

#### Histology

Mouse melanomas were formalin fixed and paraffin imbedded; hereafter cut in 4 µm slices and routinely dyed with hematoxylin and eosin. The cut was placed on tumor’s largest diameter. Tumor area was measured directly on the slide and amount of necrosis judged microscopically via overview ocular.

### Statistics

For multiple comparisons with exercise and diet, two-way ANOVA followed by post hoc tests with Bonferroni corrections were performed. Linear regression analyses were performed to describe the association between systemic factors or body weight with tumor growth. A Mann Whitney test was used to examine the γH2AX expression levels. Data analysis was performed using the GraphPad Prism version 7.0 software. Results are depicted as means ± standard error of mean (SEM). The criterion for significance was set at a probability of less than 0.05.
